# Diet and predatory behavior of the Asian ant-eating spider, *Asceua* (formerly *Doosia*) *japonica* (Araneae: Zodariidae)

**DOI:** 10.1186/s40064-016-2234-1

**Published:** 2016-05-10

**Authors:** Takashi Komatsu

**Affiliations:** The Kyushu University Museum, Hakozaki 6-10-1, Fukuoka, 812-8581 Japan

**Keywords:** Formicidae, Myrmecophagy, Red data book, Japan

## Abstract

Several spider taxa are specialized to prey on ants. Some species of Zodariidae are known to use specialized tactics to capture ants. In this study, I assessed the diet difference and predatory behavior of the Japanese zodariid *Asceua japonica*. In a series of surveys, all observed individuals in the field preyed on tiny arboreal ants representing several subfamilies. In addition, the species used tactics similar to those of its European relatives for preying on ants. This is the first observation of myrmecophagy of Zodariidae in East Asia.

## Background

Among the spiders of the world, a few are regarded as food specialists (Jackson and Hallas 1986; Jackson and Whitehouse 1986; Eberhard 1991; Dippenaar-Schoeman et al. 1996; Yeargan and Quate 1997; Platnick 2002). Ant-eating specialists are one type (Pekár 2004). Generally, ants are aggressive and have several means to resist attacks, such as biting, stinging, and using toxic chemicals (Hölldobler and Wilson 1990) so myrmecophagy is a high risk tactic for most spiders (Cushing 2012). On the other hand, a hunter that evolves strategies for overcoming an ant’s defenses and aggression faces relatively little competition for a nearly unlimited food resource (Cushing 2012).

Cushing (2012) reviewed the world’s ant-eating spider taxa and showed that over 100 species of spiders (within 14 families) were recorded to capture ants (including facultative predatory species). The author stated that the spider family with the most recorded obligatory ant-eating species is Zodariidae. Some zodariid species are known to specialize on ants, although little information is available regarding the biology of this family (Simon 1864; Wiehle 1928; Harkness 1976; Cushing and Santangelo 2002; Pekár 2009; Pekár et al. 2005, 2014). Ant-eating specialist spiders, including zodariids, have behavioral tactics to overcome ants efficiently (Pekár 2004; Cushing 2012). For example, two European zodariid species, *Zodarion germanicum* and *Z. rubidum*, skillfully prey on various ant species; making a surprise attack and retreat from the prey immediately to avoid counterattack (Pekár 2004). When they attack an ant, they always bite its legs. In the case of an Israeli species, *Z. cyrenaicum*, similar hunting behavior was observed except that juveniles sometimes bite the petiole of prey ant and keep attacking (Pekár et al. 2014). After paralysis of the prey, spiders carry it to a secluded place to feed (Harkness 1976; Cushing and Santangelo 2002; Pekár 2004). A series of these attack-and-escape tactics appear to be quite efficient for non-web-building specialist ant-eating species to capture ants, based on the fact that other families of ant-eating species also hunt in this manner (Salticidae: Jackson and Van Olphen 1992; Jackson et al. 1998; Thomisidae: Leong and D’Rozario 2012).

*Asceua* (formerly *Doosia*) *japonica* is a small zodariid (3–4 mm in body size) distributed in Taiwan and Japan (Nishikawa 2006; Shinkai 2006). Like other zodariids, information about the species’ biology is scarce because A*. japonica* is rare enough to be listed in the Red Data Book in Japan (Kishida 1940; Nishikawa 2006, 2014). Previous fragmentary observations suggest that this species is arboreal, diurnal, and makes a silk-sack for hibernation under tree bark in winter (Nishikawa 2006; Shinkai 2006). However, I have conducted preliminary field surveys and found that it preys on ants frequently. Therefore, I suspected that *A. japonica* is an ant-eating specialist that has specialized predatory tactics. Moreover, some zodariid species show distinct prey size and/or species preferences between juvenile and adult, and between male and female (Couvreur 1990; Pekár et al. 2014). It is likely that some degree of similar tendency is also recognized if *A. japonica* is truly an ant-eating specialist. In this study, I surveyed the natural diet of *D. japonica* in the field and observed its predatory behavior.

## Materials and methods

### Study area

Field surveys were conducted mainly in Tachibana-yama, Fukuoka city, Fukuoka prefecture (elevation 101 m), in conjunction with another locality in Hiranuma, Numazu city, Shizuoka prefecture (elevation 39 m), in 2014 and 2015. The latter locality was surveyed only during 1 day (28 June, 2015). Both locations were suburban secondary forests with many planted trees, and the environment was shaded and humid.

### Field survey

A preliminary survey suggested that *A. japonica* is a crepuscular species, so I conducted observations mainly in the evening (from 17:00 to 20:00), as well as during the day. In 2015, I searched for individuals exhibiting predatory behavior by looking at tree trunks from April to October. When an *A. japonica* specimen was found, I noted the body size and sex of the spider and the taxon of the prey. Body size was categorized as large (≥2 mm) and small (<2 mm). I was unable to determine the sex of small individuals in the field, so all observed individuals were classified as large male, large female, or small.

### Laboratory experiment

The field survey suggested that *A. japonica* exclusively preys on ants, so I conducted a laboratory experiment to observe the species’ predatory behavior in detail. I newly collected seven *A.japonica* individuals walking on tree trunk without prey, from Tachibana-yama (two juveniles and five adult females). Each spider was released separately into a plastic Petri dish (10 cm in diameter). A piece of creased filter paper was set in it as a shelter for the spider. Each was provided with one live ant per day. The ants represented several species that were recognized as prey in the field survey (*Camponotus vitiosus*, *Crematogaster matsumurai*, *Lasius japonicas*, *Pristomyrmex punctatus*, and *Temnothorax spinosior*). All spiders were provided the same ant species each day, and the ant species was changed from day to day.

After releasing the prey into the Petri dish, I observed the predatory behavior of the spider, particularly the following points: the body part(s) of the ant that the spider bit during capturing, and whether the spider released the captured ant or not. If the ant was not captured after 1 h, it was removed from the Petri dish.

### Statistical analysis

I determined the diet difference among different growth stage or sex of spider in the field by Fisher’s exact test. I modified two patterns of categorization for three spider groups; small versus large (male + female); large male versus large female. In this situation, I tested whether difference of prey ant species construction between small and large (or male and female) is statistically significant or not. The R software package, version 2.15.1 (http://www.R-project.org) (R Development Core Team 2012) was used.

## Results

### Field survey

During a total of 243 days (30 days in 2014 and 213 in 2015), observations of predatory behavior on 77 individuals of *A. japonica* were made (75 individuals in Fukuoka prefecture in 28 days, 2014 and 2015 and 2 individuals in Shizuoka prefecture in 1 day at June, 2015). In both areas, all of them preyed on ants without exception. The ant taxa represented 3 subfamilies, 9 genera, and 12 species, with most comprising *Pristomyrmex punctatus* (Myrmicinae), *Lasius hayashi* (Formicinae), and *Crematogaster* (=*Cre.*) *vagula* (Myrmicinae) (Table 1). Most prey species were 2–4 mm in body length, but the largest one was *Formica hayashi* (7 mm; Japanese Ant Image Database 2010). Among the three categories of *A. japonica*, large males preyed on 8 ant species, large females on 7, and small individuals on 7 (Table 2).

**Table 1:** Prey ant species and number of *Asceura japonica* that preyed on the ant (*N*) within total ant fauna in each studied areas

Subfamily	Genus	Species	Body size (mm)	Abundance	Habitat	*N*
Ant taxon (Fukuoka)						
Formicinae	*Camponotus*	*C. devestivus*	7.0–10.0	R	G–A	0
		*C. keihitoi*	4.5	R	A	0
		*C. kiusiuensis*	8.0–11.0	C	G–A	0
		*C. vitiosus*	5.0–6.0	C	A	6
	*Formica*	*F. hayashi*	5.5–7.0	C	G	1
	*Lasius*	*L. fuji*	4.0–5.0	N	G–A	0
		*L. hayashi*	2.0–4.0	C	G–A	18
		*L. japonicus*	2.5–3.5	C	G–A	1
	*Nylanderia*	*N. flavipes*	2.0–2.5	C	G	5
Myrmicinae	*Aphaenogaster*	*A. famelica*	3.5–8.0	C	G	0
	*Carebara*	*C. yamatonis*	1.0–2.0	R	G	0
	*Crematogaster*	*C. vagula*	2.0–3.0	C	A	14
		*C. matsumurai*	2.0–3.5	C	A	2
	*Lordomyrma*	*L. azumai*	3.0–4.5	R	G	0
	*Messor*	*M. aciculatus*	4.0–5.0	N	G	0
	*Monomorium*	*M. intrudens*	1.5	N	G–A	0
	*Pheidole*	*P. fervida*	2.5–3.5	C	G–A	1
		*P. noda*	3.0–4.5	C	G–A	1
	*Pyramica*	*P. benten*	1.5–2.0	R	G	0
	*Pristomyrmex*	*P. punctatus*	2.5	C	G–A	24
	*Solenopsis*	*S. japonica*	1.5	N	G	0
	*Strumigenys*	*S. lewisi*	2.0	C	G	0
	*Temnothorax*	*T.* sp.	3.0	R	G–A	1
	*Vollenhovia*	*V. emeryi*	2.5	N	G–A	0
		*V. nipponica*	2.5	R	G–A	0
Ponerinae	*Brachyponera*	*B. chinensis*	4.0	C	G–A	1
	*Hypoponera*	*H. sauteri*	2.0	N	G	0
Proceratiinae	*Discothyrea*	*D. sauteri*	2.0	R	G	0
	*Proceratium*	*P. itoi*	3.0	R	G	0
		*P. watasei*	3.5–4.0	R	G	0
Ant taxon (Shizuoka)						
Myrmicinae	*Crematogaster*	*C. matsumurai*	2.0–3.5	C	A	2

Body size of each ant species is referred to Japanese Ant Image Database (http://ant.edb.miyakyo-u.ac.jp/E//). Abundance; C: common, N: near rare, R: rare. Habitat; G: ground dweller, A: arboreal

**Table 2:** Prey species composition of *Asceura japonica* (small vs. large (male + female); large male vs. large female). Diet difference among different growth stage or sex of spider in the field by Fisher’s exact test (see in text)

Ant taxon	Spider			
	Large, Male + Female (39)		Small (38)	
	N	%	N	%
*Camponotus vitiosus*	5	13	1	3
*Formica hayashi*	1	3	0	0
*Lasius hayashi*	16	41	2	5
*Lasius japonicus*	1	3	0	0
*Nylanderia flavipes*	2	5	3	8
*Crematogaster matsumurai*	1	3	3	8
*Crematogaster vagula*	3	8	11	29
*Pristomyrmex punctatus*	7	18	17	45
*Temnothorax* sp.	1	3	0	0
*Pachycondyla chinensis*	1	3	0	0
*Pheidole fervida*	0	0	1	3
*Pheidole noda*	1	3	0	0

Ant taxon	Large, Male (10)		Large, Female (29)	
	N	%	N	%
*Camponotus vitiosus*	0	0	5	17
*Formica hayashi*	1	10	0	0
*Lasius hayashi*	2	20	14	48
*Lasius japonicus*	1	10	0	0
*Nylanderia flavipes*	1	10	1	3
*Crematogaster matsumurai*	0	0	1	3
*Crematogaster vagula*	1	10	2	7
*Pheidole noda*	0	0	1	3
*Pristomyrmex punctatus*	2	20	5	17
*Temnothorax* sp.	1	10	0	0
*Pachycondyla chinensis*	1	10	0	0

**Fig. 1:**
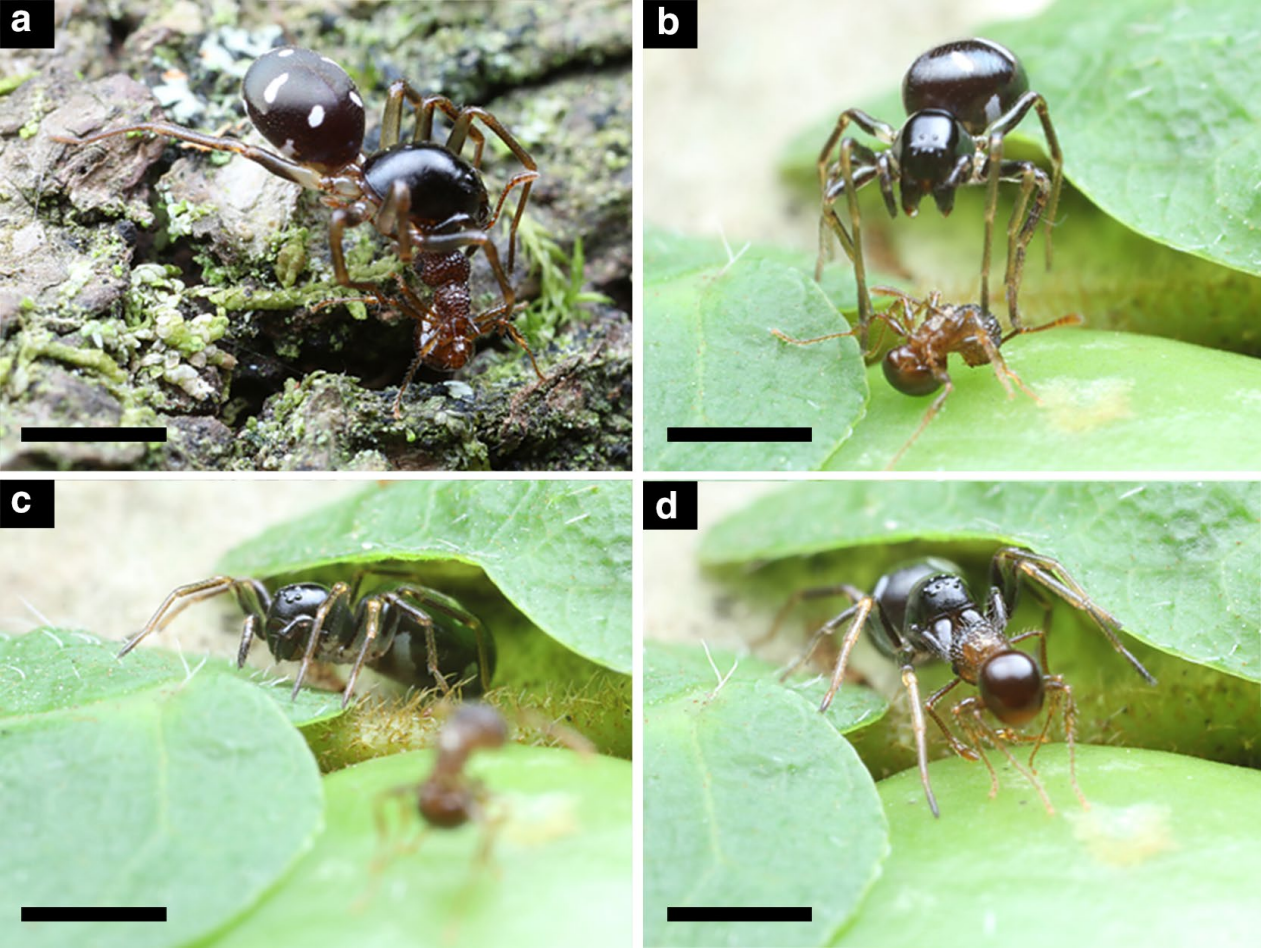
Attacking behavior of *Asceura japonica* toward *Pristmyrmex punktatus* in the field. **a** “Large Male” biting ant. **b** “Small” releasing captured ant. **c** “Small” waiting near dying ant. **d** “Small” retrieving ant

Although *A. japonica* exhibited predatory behavior during the daytime, it was most often observed in the period just before and after sunset. In 15 cases, I was able to observe the series of capturing maneuvers. An ambushing spider always lowered its eyes or turned to the side with its legs spread. Preferred ambush sites appeared to be near an ant column on a tree trunk, in a bark crevice, or behind leaves of an epiphyte like *Lemmaphyllum microphyllum*. When a spider was ambushing near an ant column, it sometimes walked along with the column as reported in *Euryopis*, other ant-eating specialist spider (Carico 1978). During the ambush phase, the spider never attacked until an approaching ant directly touched its legs. In addition, spiders only attacked ants from the front. When an ant touched the legs of the spider, it quickly placed itself over the prey (Fig. 1a). The thorax of an ant was the preferred body part for spiders to bite.

The subsequent behavior of the spider varied depending on the captured ant taxon. In the case of formicine species (e.g., *Lasius* spp., *Nylanderia flavipes*), the spider kept biting and the prey was paralyzed in a short time (about 3–4 s after biting). In the case of myrmicine species (e.g., *Crematogaster* spp., *P. punctatus*), the spider frequently released the prey at once. Especially in *P. punctatus*, all observed individuals were released in the wild (Fig. 1b). In all cases, the period of biting was 1–2 s. After releasing the prey, the spider stayed about 1 cm away from the ant with its legs closed (Fig. 1c). Although the attacked ant walked about in an agitated manner, it became paralyzed in about 10–15 s and stopped moving. At that point, the spider started flapping its forelegs as in the case of *Zodarion rubidum* (Pekár 2004) and walking slowly in a zig-zag pattern with its forelegs thrust out. It seemed to recognize the presence of the ant only when the apex of the legs touched the ant body. The spider then held the ant thorax after beating the prey with its forelegs (Fig. 1d). Regardless of the prey taxon, a spider that retrieved prey walked quickly away from the place and hid in a bark crevice or behind epiphyte leaves to consume it. If there was no hiding spot nearby, the spider consumed the prey on site.

I also observed physical contact of a mite and a springtail with ambushing *A. japonica*. In both cases, the spider attempted to bend itself toward the organism but did not capture it. In 2015, the first sighting of a *A. japonica* in Tachibana-yama was 1 May and the final date was 3 September.

### Prey species difference

I listed the diet difference among different growth stage or sex of *A. japonica* (Table 2). For comparison between small and large (male + female), composition of prey species quite significantly differentiated (Fisher’s exact test, *P* < 0.01) (Table 2). Most frequently captured ant species were *L. Hayashi* for large and *P. punctatus* for small. In addition, for comparison between large male versus large female, composition of prey species also significantly differentiated (Fisher’s exact test, *P* = 0.04) (Table 2), though most frequently captured ant species were shared by both sexes (*L. Hayashi* and *P. punctatus* for male and *L. Hayashi* for female, respectively).

### Laboratory experiment

All *A. japonica* individuals were presented with five ant species over the course of 6 days (Table 3). To determine the specificity of attacking behavior toward *P. punctatus*, I introduced this ant species a second time.

**Table 3:** Reaction toward prey ant species of *Asceura japonica* under laboratory conditions

Ant taxon	A (juvenile)	B (juvenile)	C (female)	D (female)	E (female)	F (female)	G (female)
*Temnothorax spinosior*	○	–	×	–	×	×	○
*Pristomyrmex punctatus*	○	○	–	○	○	○	–
*Lasius japonicus*	–	×	×	–	×	×	–
*Crematogaster matsumurai*	–	–	○	×	×	×	×
*Pristomyrmex punctatus*	–	–	–	○	–	○	–
*Camponotus vitiosus*	–	–	–	–	–	–	–

○: released after capturing. × : never released. –: not captured

For the myrmicine species, there was no clear pattern of predatory behavior: some spiders released these ants after capture, whereas others did not release them or did not capture them (Table 3). However, in the case of *P. punctatus*, if the ant was captured it was always released. Under laboratory conditions, the periods of biting were longer than in the wild (about 10–20 s). Tactics of attacking (whether keeping or releasing prey) was not fixed for each individual.

In the case of the formicine *L. japonicus*, if the ant was captured, none of the spiders released it. In contrast, the formicine *Camponotus* (=*Cam.*) *vitiosus* was not captured by any spider (Table 3). As in the wild, spiders attacked and bit mainly the thorax from the anterior (in a few cases, they bit the ant’s leg). When the observed spider was approached by ant from the rear, the spider escaped from the ant without preying.

## Discussion

This study revealed that *A. japonica* is a specialist that preys exclusively on small ants. All individuals observed at two geographically remote areas preyed on ants, and they used capture tactics like those of other ant-eating spiders. *Asceura japonica* cannot be regarded as a species-specific predator, because the diet consisted of ant species across several subfamilies. Nonetheless, the prey items did share some similarities. The adult size of *A. japonica* is about 3–4 mm (Shinkai 2006), and most recorded prey species were also 3–4 mm in size (Japanese Ant Image Database 2010). Large and small individuals showed a diet difference toward particular ant species (Table 2). Considering that the small spiders were <2 mm, it makes sense that they avoided *Cam. vitiosus* (with a relatively large body size) and *L. hayashi* (which moves more rapidly than other species of similar body size) as prey. Moreover, it is possible that nutrient requirements differ between juvenile and adult *A. japonica.* Pekár and Mayntz (2014) state that carbon and nitrogen is rich in Myrmicinae while lipid is rich in Formicinae in viewpoint of nutrient composition of ant as a prey.

On the other hand, although large males and females shared same ant species as most frequently captured species, composition of prey species significantly differentiated (Table 2). In studied areas, I could observe very few individual large males. To clarify whether *A. japonica* shows diet difference between males and females and between large and small body size, more extensive field surveys should be conducted. In particular, it may be important to observe prey menu of *A. japonica* before hibernation. At least one polyphagous spider species (Lycosidae: *Pardosa amentata* (Clerck, 1757)) is known to change its prey species composition before overwintering (Bressendorff and Toft 2011). In addition, lipid stores in ant bodies increase toward winter (Pekár and Mayntz 2014). It is possible that preference of prey menu for *A. japonica* vary just before winter though it remains unclear when *A. japonica* usually begins hibernation.

I conducted the field surveys mainly around sunset, because my previous observations suggested that predation of *A. japonica* was easily seen in the evening. I also visited a field site during daytime and could find only a few individuals exhibiting predatory behavior. *Asceura japonica* has been regarded as a diurnal species (Shinkai 2006), though both active and resting individuals were seen at any time of day or night in Tachibana-yama. These observations suggest that *A. japonica* wonder on tree trunk not only at daytime but also at night. On the other hand, it tends to capture prey mainly from dusk to after sunset.

*Asceura japonica* changes its attacking tactic depending on the prey. This may indicate that the efficacy of the venom varies among different ant species, as Pekár (2004) suggested. My observations indicated that more time was needed to paralyze myrmicine prey than formicine prey. It is interesting to consider how spiders judge an ant taxa at the moment of capture. The use of visual cues seems unlikely, as they do not appear to have extraordinary eyesight relative to other taxa of spiders. For example, individuals did not attack prey without physical contact, and they did not directly approach paralyzed prey during retrieval. Therefore, *A. japonica* may use a chemical cue like the cuticular hydrocarbon composition for determining ant taxon. *Siler* sp., another Japanese ant-eating spider, also changes tactics depending on prey taxon. It tends to prefer attacking workers of larger ant species, but selects larvae or pupae carried by workers in an ant column for smaller ant species (Sato et al. 2012). In the case of *Siler* sp. (Salticidae) (Karsch 1879), visual cues may be fundamental for judging the ant taxon because it has excellent eyesight, a trait specific to Salticidae.

Although the laboratory experiment also confirmed that *A. japonica* changes tactics depending on the prey taxon, there were some irregular reactions that were not seen in the wild. In attacking *P. punctatus*, all spiders bit the prey for a longer period than in wild. In addition, *Cam. vitiosus* was selected as prey in the wild but was avoided in the laboratory. Throughout the laboratory experiment, the spiders were relatively inactive toward predation. The reason remains unclear, but reception of an ant trail pheromone may work as a cue to encourage *A.japonica* to transition to normal ambush behavior. European zodariid species, *Z. rubidum*, is known to able recognize chemical cues from trail pheromone of specific ant genera (Cárdenas et al. 2012).

The manner of attack of *A. japonica* is slightly different from that of other known zodariids. European species bite ant legs from the rear (Pekár 2004), whereas most individuals of *A.* *japonica* bit the ant’s thorax from the anterior. It is interesting that different predatory tactics exist within Zodariidae. Given that another ant-eating taxon, *Callilepis* (Gnaphosidae), attacks from the anterior but bites only the ant’s antennae (Heller 1974), even more tactics may exist within Zodariidae.

## Conclusion

*Asceura japonica* is regarded as a rare spider whose distribution is restricted, but the species’ diet is composed of a variety of common ants that are widely distributed. Therefore, several factors other than food may limit the species’ regional distribution, such as humidity, temperature, and sunshine. In this study, both observed areas were dark evergreen forests where huge trees form stands, although residential areas were nearby. The trunk surface of all the huge trees (about >30 cm and >15 m in diameter and tree height, respectively) in the forest were covered by epiphytes. Further research is needed to clarify the species’ true distribution and which environmental factors restrict its range.

In addition, this study suggests that various tactics to overcome prey have evolved within Zodariidae and even among other ant-eating spider taxa. Field observations, biochemical analyses, and ant neurological studies may provide profound insights into the interaction between ants and ant-eating spiders.
